# Frailty and hospitalization-associated disability after pneumonia: A prospective cohort study

**DOI:** 10.1186/s12877-021-02049-5

**Published:** 2021-02-05

**Authors:** Chan Mi Park, Wonsock Kim, Hye Chang Rhim, Eun Sik Lee, Jong Hun Kim, Kyung Hwan Cho, Dae Hyun Kim

**Affiliations:** 1 Department of Family Medicine, Korea University Anam Hospital, Korea University College of Medicine, Seoul, Republic of Korea; 2 Harvard T.H Chan School of Public Health, Boston, MA USA; 3 Division of Infectious Diseases, Department of Internal Medicine, CHA Bundang Medical Center, CHA University, 59 Yatap-ro, Bundang-gu, Seongnam 13496 Republic of Korea; 4 Hinda and Arthur Marcus Institute for Aging Research, Hebrew SeniorLife, Harvard Medical School, Boston, MA USA

**Keywords:** Frailty, Pneumonia, Disability, Hospitalization‐associated disability

## Abstract

**Background:**

Pneumonia is a major cause of morbidity and mortality in older adults. The role of frailty assessment in older adults with pneumonia is not well defined. Our purpose of the study was to investigate 30-day clinical course and functional outcomes of pneumonia in older adults with different levels of frailty.

**Methods:**

A prospective cohort was conducted at a university hospital in Seoul, Korea with 176 patients who were 65 years or older and hospitalized with pneumonia. A 50-item deficit-accumulation frailty index (FI) (range: 0–1; robust < 0.15, pre-frail 0.15–0.24, mild-to-moderately frail 0.25–0.44, and severely frail ≥ 0.45) and the pneumonia severity CURB-65 score (range: 0–5) were measured. Primary outcome was death or functional decline, defined as worsening dependencies in 21 daily activities and physical tasks in 30 days. Secondary outcomes were intensive care unit admission, psychoactive drug use, nasogastric tube feeding, prolonged hospitalization (length of stay > 15 days), and discharge to a long-term care institution.

**Results:**

The population had a median age 79 (interquartile range, 75–84) years, 68 (38.6 %) female, and 45 (25.5 %) robust, 36 (47.4 %) pre-frail, 37 (21.0 %) mild-to-moderately frail, and 58 (33.0 %) severely frail patients. After adjusting for age, sex, and CURB-65, the risk of primary outcome for increasing frailty categories was 46.7 %, 61.1 %, 83.8 %, and 86.2 %, respectively (*p* = 0.014). The risk was higher in patients with frailty (FI ≥ 0.25) than without (FI < 0.25) among those with CURB-65 0–2 points (75 % vs. 52 %; *p* = 0.022) and among those with CURB-65 3–5 points (93 % vs. 65 %; *p* = 0.007). In addition, patients with greater frailty were more likely to require nasogastric tube feeding (robust vs. severe frailty: 13.9 % vs. 60.3 %) and prolonged hospitalization (18.2 % vs. 50.9 %) and discharge to a long-term care institution (4.4 % vs. 59.3 %) (*p* < 0.05 for all). Rates of intensive care unit admission and psychoactive drug use were similar.

**Conclusions:**

Older adults with frailty experience high rates of death or functional decline in 30 days of pneumonia hospitalization, regardless of the pneumonia severity. These results underscore the importance of frailty assessment in the acute care setting.

## Background

Pneumonia is a major cause of morbidity and mortality in older adults [1]. In the United States, 5.6 million new cases are reported every year, and it accounts for high mortality and annual medical costs exceeding $10 billion [2]. In Asia, pneumonia is responsible for almost 1 million adult deaths every year [3]. In Japan, pneumonia affected 1.8 million older Japanese and was the fifth leading cause of death in 2017 [4]. In Korea, it ranked fourth leading cause of death with a mortality rate of 11.6 % [5, 6] and the annual medical costs of $400 million [6, 7].

Previous studies suggest that many patients with pneumonia have persistent symptoms and poor physical health after 30 days of diagnosis [8, 9]. Older adults are particularly at high risk for poor outcomes [10], but the determinants of poor outcomes that are specific to this population have not been well characterized. Clinical risk stratification tools, such as CURB-65 [11] or Pneumonia Severity Index [9], predict mortality based on demographic information, comorbidities, or physiological parameters. However, these tools do not consider frailty—a clinical state of reduced physiologic reserve and increased vulnerability to poor health outcomes [12]—that is germane to clinical management of older adults. The prevalence of frailty ranges from 10 % in the community [13] to more than 50 % in the nursing homes [14] and hospitals [15], and it has been associated with poor health outcomes in the primary care [16], acute hospital [17], and critical care setting [18]. Therefore, assessing frailty on admission may provide information about patients’ vulnerability and prognosis that is not captured by the pneumonia severity and is useful to deliver patient-centered care to improve recovery.

We conducted a prospective cohort study to evaluate the association of frailty with 30-day clinical and functional outcomes in older adults hospitalized with pneumonia. We hypothesized that frailty assessed on admission would be associated with mortality or functional decline at 30 days, independently of a pneumonia-specific risk score. We also examined key care process measures during the acute hospitalization.

## Methods

### Study design and population

**Fig. 1:**
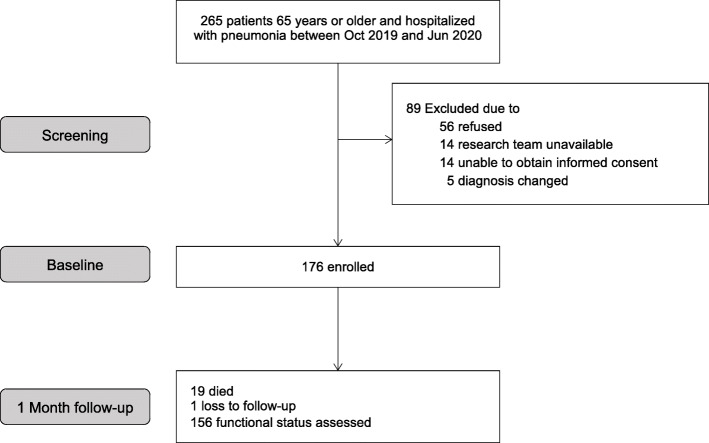
Selection of Study Population

This prospective cohort study was approved by the Institutional Review Board and written informed consent was obtained from all patients or their proxy. Between October 2019 and June 2020, we approached consecutive patients who were 65 years or older and hospitalized with pneumonia at University Hospital, Seoul, Korea (see Flow Diagram in Fig. 1). Pneumonia was diagnosed based on symptoms (e.g., fever, cough, sputum, and dyspnea) plus an infiltrate on chest radiograph. During our study period, patients diagnosed with the novel Coronavirus Disease 2019 (COVID-19) were transferred to government-designated hospitals and only those with negative COVID-19 tests were admitted to our hospital. Among 265 patients screened, 89 were excluded for the following reasons: (1) patient declined (*n* = 56); (2) research team was unavailable (*n* = 14); (3) informed consent could not be obtained from the patient or his/her proxy (*n* = 14); and (4) change in diagnosis after admission (*n* = 5). Finally, 176 patients were included in our study.

### Baseline assessment

Study clinicians (CMP, WK, ESL) evaluated patients for medical comorbidities, self-reported functional status, cognitive function, nutritional status, and social support on admission. Self-reported functional status 30 days prior to admission was assessed by asking patients or their proxy about the ability to perform 21 daily activities and physical tasks without another person’s assistance: 7 activities of daily living (feeding, dressing, grooming, ambulating, transferring, bathing, and toileting), 7 instrumental activities of daily living (doing housework, making telephone calls, using transportation or driving, shopping, cooking, taking medications, and managing money), and 7 activities in the Nagi and Rosow-Breslau scales (pulling or pushing a large object, lifting 5 kg, walking up and down a flight of stairs, walking 1 km, writing or handling small objects, reaching arms above shoulder, and stooping, crouching, or kneeling) [19, 20]. Mobility impairment was defined as inability to ambulate in the house, walk 1km, or walk up and down a flight of stairs. Cognition was assessed using Mini-Mental State Examination (MMSE) or the AD8 questionnaire [21] if a patient was unable to participate in MMSE. Cognitive impairment was defined as having a dementia diagnosis, MMSE < 19 points [22], or AD8 ≥ 3 points [21]. Study clinicians (CMP, HCR) reviewed medical records to extract sociodemographic characteristics, admission source (nursing home vs. home), medical comorbidities, vital signs, body mass index, and laboratory test results (e.g., serum albumin).

### Measurements of frailty, pneumonia severity, and comorbidity burden

A deficit-accumulation frailty index (FI) [23] was calculated using 50 items from baseline assessment: 26 comorbidities, polypharmacy (≥ 5 prescription drugs), self-reported ability to perform 21 activities listed above, weight loss > 5 kg in past year, body mass index < 21 kg/m^2^, and serum albumin < 3.5 g/L [24, 25]. MMSE was excluded from the FI calculation due to low completion rate (38.6 %). Based on FI (range: 0–1), patients were classified into robust (< 0.15), pre-frail (0.15–0.24), mild-to-moderately frail (0.25–0.44), and severely frail (≥ 0.45) categories. Pneumonia severity was calculated using CURB-65 score (range: 0–5), which includes confusion, uremia, elevated respiratory rate, hypotension, and ≥ 65 years of age [11]. The Gagne comorbidity index (range: 0–24) was used to quantify the comorbidity burden [26].

### Outcome assessment

At 1 month after baseline assessment, study clinicians (CMP, WK) conducted telephone interviews with patients or their proxy to assess self-reported functional status. Except 1 patient who was lost to follow-up and 19 patients who died, we were able to interview 156 patients. A disability score (range: 0–21) was calculated as the total number of activities requiring another person’s assistance. Primary outcome was a composite endpoint of death or functional decline, defined as any increase in the disability score between baseline and 1 month. As secondary patient outcomes, we examined death and functional decline at 30 days separately. As secondary process outcomes, we assessed intensive care unit (ICU) admission, psychoactive drug (antipsychotics, benzodiazepines, or hypnotics) use, nasogastric tube feeding, prolonged hospitalization (length of stay > 15 days), and discharge to long-term care institution.

### Statistical analysis

We compared baseline characteristics of patients across frailty categories using chi-square test for categorical variables and analysis of variance or Kruskal-Wallis test for continuous variables. The risks of primary and secondary outcomes were compared across frailty categories using chi-square test and logistic regression to estimate the odds ratio (OR) and 95 % confidence interval (CI) adjusting for age, sex, and CURB-65 score (3–5 vs. 0–2 points). Patients with the maximum disability at baseline were excluded from analysis for primary outcome and functional decline. Those who were admitted from a nursing home were excluded from analysis for long-term care institutionalization. We also examined the prevalence of each disability at baseline and 30 days later by frailty level on admission. To illustrate the importance of pneumonia severity and frailty, we estimated age- and sex-adjusted risk of primary outcome by CURB-65 (3–5 vs. 0–2 points) and frailty category (frail [FI ≥ 0.25] vs. non-frail [FI < 0.25]) from a logistic model that included age, sex, CURB-65, frailty category, and the interaction term between CURB-65 and frailty categories. Lastly, in order to test the association between FI and CURB-65, spearman’s correlation was calculated. Analysis was performed using Stata version 16 (StataCorp, LLC, College Station, Texas). A 2-sided *p*-value < 0.05 was considered statistically significant.

## Results

### Characteristics of study population

The FI ranged from 0.02 to 0.65, with 45 (25.5 %) robust, 36 (47.4 %) pre-frail, 37 (21.0 %) mild-to-moderately frail, and 58 (33.0 %) severely frail patients (Table 1). The study population had a median age of 79 (interquartile range [IQR], 75–84) years, 68 (38.6 %) female, and 35 (19.9 %) patients admitted from a nursing home. Generally, patients with greater frailty levels were older (median age [IQR] for robust vs. severe frailty: 77 [73–82] vs. 81 [75–84] years), admitted from a nursing home (0 [0 %] vs. 31 [53.5 %]), and have more severe pneumonia (CURB-65 score 3–5 points: 10 [22.2 %] vs. 36 [62.1 %]) and higher comorbidity burden (median Gagne index [IQR]: 2 [1,2,3] vs. 4 [3,4,5,6]). Increasing frailty was associated with stroke (robust vs. severe frailty: 8 [17.8 %] vs. 28 [48.3 %]), disability (ADL: 0 [0 %] vs. 58 [100 %] and IADL: 3 [6.7 %] vs. 58 [100 %]), mobility impairment (7 [15.6 %] vs. 58 [100 %]), and cognitive impairment (7 [15.6 %] vs. 58 [100 %]) and lower body mass index (mean [standard deviation]: 22.4 [3.6] vs. 18.6 [3.8] kg/m^2^). The spearman’s correlation between FI and CURB-65 was 0.34; *p* < 0.001, indicating a weak positive relationship.

**Table 1:** Characteristics of Older Patients Hospitalized with Pneumonia

Characteristics	Total	Frailty Category				***P*** value
		Robust	Pre-frailty	Mild-to-moderate frailty	Severe frailty	
Sample Size	176	45	36	37	58	NA
Frailty Index, median (IQR)	0.31 (0.14, 0.50)	0.10 (0.06, 0.12)	0.18 (0.16, 0.20)	0.35 (0.31, 0.41)	0.54 (0.50, 0.58)	<0.001
Age, years, median (IQR)	79 (75, 84)	77 (73, 82)	80 (74, 84)	81 (77, 86)	81 (75, 84)	0.035
Female, n (%)	68 (38.6)	14 (31.1)	14 (38.9)	17 (46.0)	23 (39.7)	0.586
Nursing Home Resident, n (%)	35 (19.9)	0 (0)	1 (2.8)	3 (8.1)	31 (53.5)	<0.001
CURB-65 Score 3-5	81 (46.0)	10 (22.2)	14(38.9)	21 (56.8)	36 (62.1)	<0.001
Gagne Index, median (IQR)	3 (1, 4)	2 (1, 3)	2 (1, 4)	3 (1, 5)	4 (3, 6)	<0.001
Cardiovascular Disease, n (%)	48 (27.3)	11 (24.4)	9 (25.0)	14 (37.8)	14 (24.1)	0.450
Diabetes, n (%)	64 (36.4)	12 (26.7)	12 (33.3)	17 (46.0)	23 (39.7)	0.294
COPD, n (%)	28 (15.9)	6 (13.3)	8 (22.2)	9 (24.3)	5 (8.6)	0.135
Stroke, n (%)	49 (27.8)	8 (17.8)	3 (8.3)	10 (27.0)	28 (48.3)	<0.001
ADL Dependency, n (%)	84 (47.7)	0 (0.0)	2 (5.6)	24 (64.9)	58 (100.0)	<0.001
IADL Dependency, n (%)	107 (60.8)	3 (6.7)	10 (27.8)	36 (97.3)	58 (100.0)	<0.001
Mobility Impairment, n (%)	124 (70.5)	7 (15.6)	24 (66.7)	35 (94.6)	58 (100.0)	<0.001
Cognitive Impairment, n (%)	132 (75.0)	37 (82.2)	21 (58.3)	26 (70.3)	48 (82.8)	0.031
BMI, kg/m^2^, median (IQR)	22.0 (4.6)	22.4 (3.6)	24.7 (5.1)	22.0 (3.9)	18.6 (3.8)	<0.001
Weight Loss, n (%)	42 (23.9)	8 (17.8)	5 (13.9)	10 (27.0)	19 (32.8)	0.132

*Abbreviations*: *ADL* activities of daily living, *BMI* body mass index, *COPD* chronic obstructive pulmonary disease, *IADL* instrumental activities of daily living, *IQR* interquartile range

### Frailty and patient outcomes at 30 days

Among 147 patients who did not have the maximum disability on admission, 99 (67.4 %) developed the primary outcome (19 deaths and 84 with functional decline). The risk of primary outcome increased with the frailty level on admission, which remained statistically significant after adjusting for age, sex, and CURB-65 (Table 2): 46.7 % for robust group, 61.1 % for pre-frail group (adjusted OR [95 % CI], 1.46 [0.58–3.69]), 83.8 % for mild-to-moderate frailty group (3.95 [1.31–11.89]) and 86.2 % for severe frailty group (5.34 [1.54–18.49]). Similar patterns were observed for the individual outcomes of death and functional decline, but the association was not statistically significant for death due to low event rates.

**Table 2:** Frailty and Outcomes in Older Patients Hospitalized with Pneumonia

Outcomes	Number of Outcome Events (%) and OR (95% CI)^**a**^					***P*** value
	Total	Robust	Pre-frailty	Mild-to-moderate frailty	Severe frailty	
**Primary Outcome**						
Death or functional decline at 30 days^b^	99 (67.4)	21 (46.7)	22 (61.1)	31 (83.8)	25 (86.2)	<0.001
	NA	Reference	1.46 (0.58-3.69)	3.95 (1.31-11.89)	5.34 (1.54-18.49)	0.014
**Secondary patient outcomes**						
Death at 30 days	19 (10.8)	3 (6.7)	1 (2.8)	6 (16.2)	9 (15.5)	0.129
	NA	Reference	0.38 (0.37-3.84)	2.36 (0.51-10.94)	2.27 (0.54-9.62)	0.281
Functional decline at 30 days^b^	84 (63.6)	18 (42.9)	21 (60.0)	25 (80.7)	20 (83.3)	0.001
	NA	Reference	1.63 (0.63-4.20)	3.84 (1.24-11.86)	5.33 (1.50-19.02)	0.022
**Secondary process outcomes**						
Intensive care unit stay	38 (21.8)	4 (8.9)	8 (22.2)	9 (24.3)	17 (30.4)	0.074
	NA	Reference	2.51 (0.67-9.44)	2.44 (0.64-9.27)	3.15 (0.92-10.73)	0.336
Psychoactive drug use^c^	41 (26.5)	10 (22.2)	7 (22.6)	13 (41.9)	11 (22.9)	0.189
	NA	Reference	0.88 (0.28-2.78)	1.92 (0.64-5.78)	0.66 (0.22-1.96)	0.236
Nasogastric tube feeding	50 (28.4)	3 (6.7)	5 (13.9)	7 (18.9)	35 (60.3)	<0.001
	NA	Reference	1.97 (0.42-9.22)	2.50 (0.56-11.22)	17.08 (4.49-64.99)	<0.001
Prolonged hospitalization (≥15 days)	62 (35.6)	8 (18.2)	14 (38.9)	11 (29.7)	29 (50.9)	0.006
	NA	Reference	2.72 (0.95-7.82)	1.58 (0.52-4.80)	3.72 (1.38-9.98)	0.039
Discharge to a long-term care institution^d^	39 (27.7)	2 (4.4)	7 (20.0)	14 (41.2)	16 (59.3)	<0.001
	NA	Reference	4.56 (0.83-24.87)	9.79 (1.88-51.00)	25.11 (4.70-134.07)	<0.001

*Abbreviations*: *CI* confidence interval, *NA* not applicable, *OR* odds ratio

^a^Adjusted for age, sex, and CURB-65 score

^b^Assessed for 147 patients who did not have the maximum disability on admission

^c^Assessed for 155 patients who were not receiving psychoactive drugs on admission

^d^Assessed for 141 patients who were not admitted from a nursing home

**Fig. 2:**
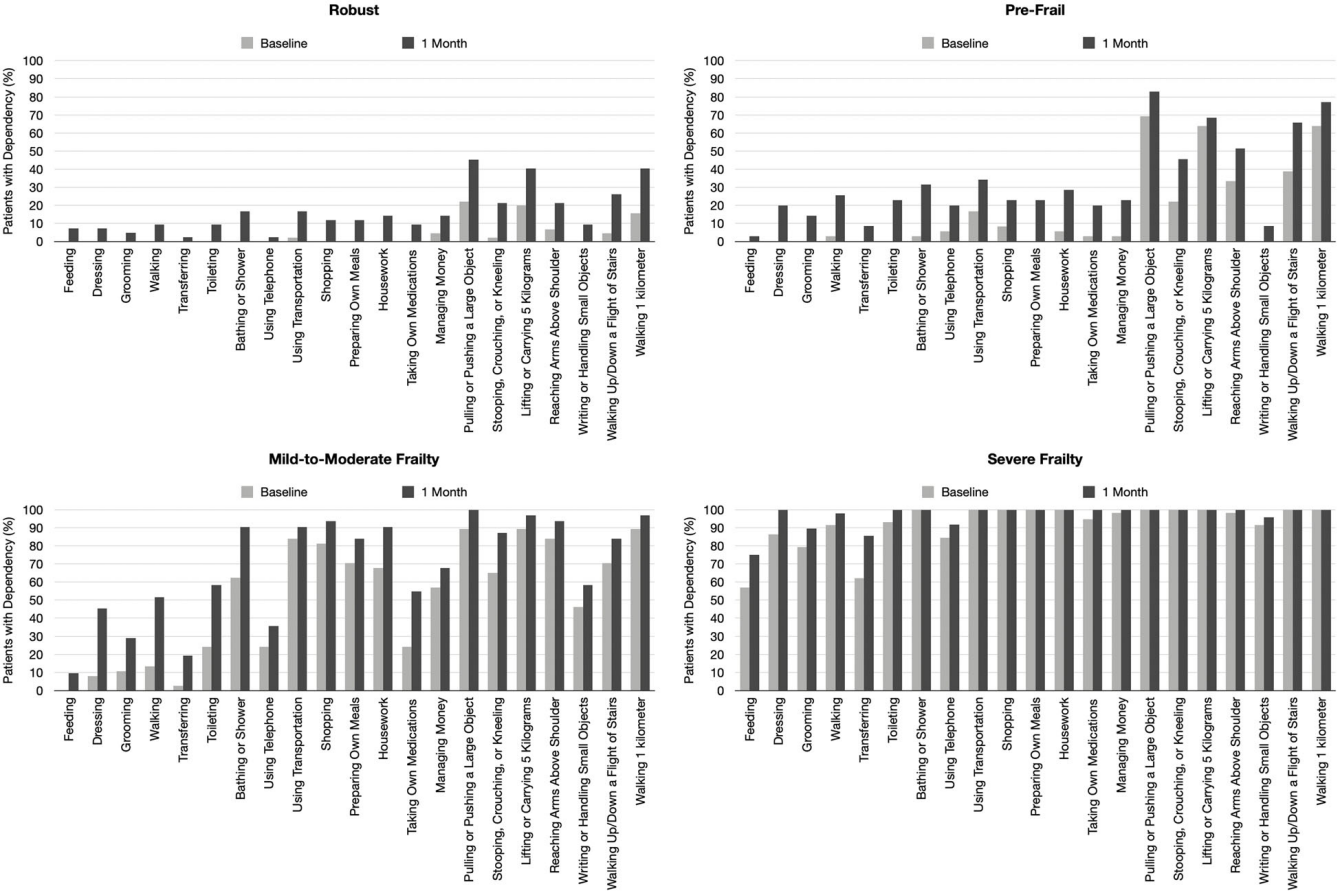
Prevalence of Disability Before and After Pneumonia Hospitalization by Frailty Level on Admission

The prevalence of each disability generally increased over 30 days across all frailty categories, except for severely frail patients who had high prevalence of pre-existing disabilities (Fig. 2). At 30 days, a large proportion of patients were unable to walk 1 km (from robust to severely frail category: 40.5 %, 77.1 %, 96.8 %, and 100.0 %) or walk up and down a flight of stairs (26.2 %, 65.7 %, 83.9 %, 100.0 %). Many patients needed help with ADLs, such as bathing or shower (from robust to severely frail category: 16.7 %, 31.4 %, 90.3 %, and 100.0 %) and toileting (9.5 %, 22.9 %, 58.1 %, 100.0 %).

**Fig. 3:**
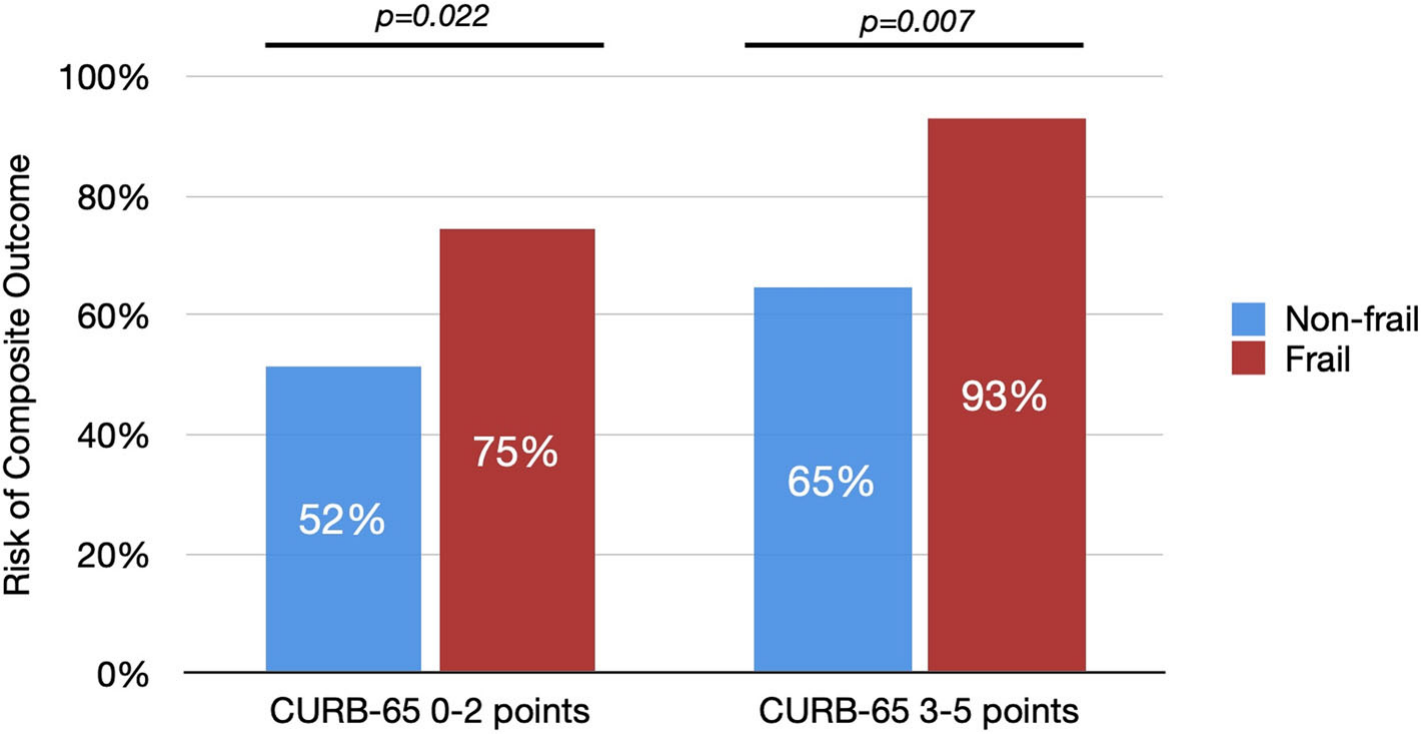
Risk of Death or Functional Decline at 30 days by Frailty and Pneumonia Severity. The risk (vertical bar) of death or functional decline was presented for frail (red bar; frailty index ≥0.25) vs non-frail (blue bar; frailty index <0.25) patients with low pneumonia severity (CURB-65 0-2 points) vs high pneumonia severity (CURB-65 3-5 points)

When we examined the risk of death or functional decline at 30 days by frailty level and pneumonia severity determined using CURB-65 on admission (Fig. 3), we found that frail patients had higher risk than non-frail patients among those with low CURB-65 scores (75 % vs. 52 %; *p* = 0.022) and among those with high CURB-65 scores (93 % vs. 65 %; *p* = 0.007). The interaction between frailty level and CURB-65 category was not statistically significant on the multiplicative scale (*p* = 0.312), which means that the association of frailty with the primary outcome was constant across the pneumonia severity category.

### Frailty and process outcomes

After adjusting for age, sex, and CURB-65, patients with greater frailty were more likely to require nasogastric tube feeding (robust vs. severe frailty: 5 [13.9 %] vs. 35 [60.3 %]) and prolonged hospitalization (8 [18.2 %] vs. 29 [50.9 %]) and be newly discharged to a long-term care institution (2 [4.4 %] vs. 16 [59.3 %]) (Table 2). However, new psychoactive drug use or ICU stay did not significantly differ by frailty level after adjustment.

## Discussion

In our prospective cohort of 176 older patients hospitalized with pneumonia, frailty on admission was associated with death or functional decline at 30 days, independently of the pneumonia severity. In particular, 9 out of 10 patients with frailty and high CURB-65 score and 3 out of 4 patients with frailty and low CURB-65 score died or experienced functional decline at 30 days. The risk of nasogastric tube insertion, prolonged hospitalization, and new long-term care institutionalization was also higher for frail patients. These findings underscore the importance of assessing frailty, in addition to the pneumonia severity, to accurately inform patients and their family about prognosis and the ongoing care needs after a pneumonia hospitalization.

It is well accepted that older adults with frailty are more vulnerable than non-frail patients to the negative effect of acute stressors [12, 27], yet little is known about the relationship of frailty, severity of acute illness, and functional recovery after an acute illness [27]. A few studies have examined the association of frailty with clinical outcomes, such as mortality, ICU length of stay and readmission rates in the setting of acute illness [28, 29]. However, these studies did not assess functional status, which is a key determinant of older adults’ independence and quality of life after hospitalization [30]. Moreover, the inter-relationship between frailty and the severity of acute illness on outcomes has not been fully explored. Although CURB-65 score predicts 30-day mortality and helps decisions about the level of care [11], recent studies reported its suboptimal performance for older adults [31, 32], particularly those 85 years or older [33]. Our study builds upon these previous studies by showing that both FI and pneumonia severity are important predictors of poor clinical and functional outcomes in older adults with pneumonia.

The current study adds to the literature on the feasibility of measuring frailty in the acute care setting. In previous studies of hospitalized patients, Clinical Frailty Scale (CFS), FI, and frailty phenotype were commonly used [27]. In the acute care setting, the simple CFS, and the 5-item FRAIL questionnaire were used more often than FI and frailty phenotype, which typically required more time and modifications to the original definition [34–36]. Although simpler measures of frailty may be useful for rapid detection of who might be frail, a more sophisticated tool, such as comprehensive geriatric assessment (CGA)-based FI, should be followed to assess and confirm the severity of frailty for clinical management [37]. In our study, FI assessment and calculation took approximately 30–45 min at the patient’s bedside with few missing items. While this may be considered time-consuming, use of routinely available electronic medical record or administrative claims data [38, 39], online calculators [25, 40], or mobile applications [41] may shorten administration time and improve interpretation.

Moreover, our results underscore high incidence of hospitalization-associated disability in older adults after a pneumonia hospitalization. Such hospitalization-associated disability occurs frequently among older adults after acute illness and sometimes they are irreversible [42–44]. Our study and several others [17, 45, 46] have shown that CGA can identify older adults at high risk for hospitalization-associated disability. Hospitalized older adults who received clinical care based on CGA were more likely to live at home and less likely to be admitted to a nursing home [45, 47]. The assessment is an essential first step to identify frail patients but it also works as an intervention by delivering individualized treatments to patients [17, 47]. According to our findings, preventative interventions for older patients with frailty should begin during the hospitalization period to avoid functional decline and promote independence. These interventions may include regular ambulation, encouraging performance of activities of daily living, and education for patients or their caregivers to assess noticeable change in functional status.

Major strengths of our study include prospective evaluation of a deficit-accumulation FI in the acute care setting, measurement of pneumonia severity, and high rates of 30-day follow-up assessment for functional status. Our study has a few limitations that deserve mention. First, the rates of death or functional decline in our cohort of older Koreans may not be generalizable to other populations in different health systems. Second, functional status was self-reported, rather than direct observation. However, the validity of self-reported functional status has been demonstrated [48], and we believe that telephone interview may be the most practical method to attain high response rates, especially during COVID-19 pandemic. Third, we only assessed outcomes over 30 days. Longer follow-up assessments are underway. Lastly, our frailty assessment did not include physical performance test and MMSE due to low completion rates in the acute care setting. Despite this missingness, we found that our FI was associated with the primary outcome. According to a recent study, missing clinical domains may affect the prevalence estimates of frailty but have little impact on the predictive ability [49].

## Conclusions

In conclusion, our study shows high rates of death or functional decline at 30 days in older patients with frailty hospitalized with pneumonia, regardless of the pneumonia severity. Frailty assessment in the acute care setting is feasible and useful to accurately inform prognosis and care needs following hospitalization. Further research is warranted to test acute care and post-acute care interventions to prevent functional decline after pneumonia.

## Data Availability

The datasets used and/or analyzed during the current study are available from Dr. Chan Mi Park and Dr. Jong Hun Kim on reasonable request.

## Abbreviations

FI: Frailty Index
OR: Odds ratio
CI: Confidence interval
CFS: Clinical frailty scale
CGA: Comprehensive geriatric assessment
